# Plasma fibrinogen, d-dimer, and fibrin degradation product as biomarkers of rheumatoid arthritis

**DOI:** 10.1038/s41598-021-96349-w

**Published:** 2021-08-19

**Authors:** Li Xue, Li Tao, Xueyi Li, Yan Wang, Biao Wang, Yanping Zhang, Ning Gao, Yanying Dong, Nan Xu, Chaoliang Xiong, Ting Zhou, Zeshi Liu, Hailong Liu, Juntao He, Ke Li, Yan Geng, Ming Li

**Affiliations:** 1 Department of Clinical Laboratory, The Second Affiliated Hospital of Medical College of Xi’an Jiaotong University, Xi’an, 710004 China; 2 Department of Rheumatology, The Second Affiliated Hospital of Xi’an Jiaotong University, Xi’an, 710061 China; 3 Department of Immunology and Pathogenic Biology, Health Science Center, Xi’an Jiaotong University, Xi’an, 710061 China; 4 Core Research Laboratory, The Second Affiliated Hospital of Xi’an Jiaotong University, Xi’an, China; 5 National Local Joint Engineering Research Centre of Biodiagnostics and Biotherapy, The Second Affiliated Hospital of Xi’an Jiaotong University, Xi’an, China; 6 Department of Cardiovascular Surgery, The First Affiliated Hospital of Xi’an Jiaotong University, Xi’an, 710061 China

**Keywords:** Medical research, Rheumatology

## Abstract

This study aimed to assess the association of coagulation-related indicators such as plasma fibrinogen (FIB), d-dimer, and fibrin degradation product (FDP) in rheumatoid arthritis (RA) with the disease activity. Data from 105 RA patients and 102 age- and gender-matched healthy controls were collected in the retrospective study. Disease activity score in 28 joints based on C-reactive protein (DAS28-CRP) was used to divide RA patients into low activity group (DAS28-CRP ≤ 2.7) and active group (DAS28-CRP > 2.7). Receiver operating characteristic (ROC) curve was applied to determine area under the curve (AUC). The association between plasma FIB, d-dimer, and FDP and DAS28-CRP was evaluated by spearman correlation. Logistical regression analysis was used to identify the independent variables associated with RA disease activity. RA patients showed higher levels of plasma FIB, d-dimer, and FDP than the controls (*P* < 0.01). Plasma FIB, d-dimer, and FDP were also increased in active groups of RA patients than those in inactive groups (*P* < 0.001). ROC curve analyses revealed that the AUC of d-dimer was higher than erythrocyte sedimentation rate (ESR) and rheumatoid factor (RF), and that of FDP was higher than RF in RA patients. In addition, the optimal cut-off value of plasma FIB, d-dimer, and FDP for RA diagnosis was 286 mg/dL, 470 μg/L, and 1.45 mg/L, respectively. Spearman analysis showed that plasma FIB, d-dimer, and FDP were positively related with DAS28-CRP (*P* < 0.001) in RA patients. Logistical regression analysis showed that d-dimer (odds ratio 2.862, 95% confidence interval 1.851–5.426, *P* < 0.001) was an independent variable associated with RA disease activity. FIB, d-dimer, and FDP were increased in RA patients and positively correlated with the disease activity of RA. d-dimer may act as a novel inflammatory indice for indicating disease activity in RA patients.

## Introduction

Rheumatoid arthritis (RA) is a systemic autoimmune disease characterized by inflammation and proliferation of synovium, progressive destruction of articular cartilage and bone, which eventually lead to severe disability, systemic complications and increased risk of mortality^1,2^. The estimated prevalence of RA is approximately 0.5–1% in adults^3^. With accumulating effective biologics applied to RA treatment, early diagnosis and accurate assessment of disease activity are absolutely urgent^4^. However, present laboratory parameters, including erythrocyte sedimentation rate (ESR), C-reactive protein (CRP), rheumatoid factor (RF), and anti-cyclic citrullinated peptide (anti-CCP) are not enough for early diagnosis^5^. By using newly developed technologies, it is possible to find and incorporate more sensitive and specific biomarkers into clinical practice of RA.

Recently it is regarded that immune and coagulation systems are functionally connected^6,7^. Pro-inflammatory cytokines have been described to be responsible for activating coagulation factors and down-regulating several important physiologic anticoagulant pathways^8–11^. A previous study revealed that IL-6 could mediate thrombocytosis, platelet hyperreactivity, and accelerate extra-intestinal thrombosis associated with experimental colitis^12^. Another study showed that T-cell-dependent IL-6 signaling was involved in angiotensin II-induced thromboinflammation^13^. As one of the most important members in the coagulation system, tissue factor could also mediate the production of pro-inflammatory cytokines through activating protease-activated receptors on various types of cells such as mononuclear cells, endothelial cells, platelets, and so on^14^. Based on these findings, several studies demonstrated the important role of coagulation-related indices in assessing the disease activity of autoimmune diseases^15^.

It has been recognized that autoimmune diseases such as RA, ankylosing spondylitis, and lupus nephritis were associated with the disorder of coagulation system^16–18^. There were studies showing that coagulation was activated in the joint of RA patients^16,19^, and the alteration in levels of coagulation factors has been related to vascular diseases present in RA^20^. Fibrinogen (FIB) is a key factor implicated in the process of blood coagulation cascade, and its deposition in the joint was identified to be characteristic of RA and even may be responsible for the formation of pannus tissue^16^. However, little is known about the predictive role of peripheral blood FIB in RA patients. As one kind of fibrin degradation products (FDPs), d-dimer is the most frequently used indicator to reflect the activation of the coagulation system.

d-dimer and other FDPs could also affect the release of inflammatory cytokines by promoting the activation of monocyte^21,22^. Although the level of d-dimer was reported to be increased in the synovial fluid of RA patients compared with osteoarthritis patients^16^, there were few studies reporting the association between the level of d-dimer in peripheral blood and the disease activity of RA patients. The aim of this study was to investigate the correlation of coagulation-related indices including plasma FIB, d-dimer, and FDP with the disease activity in RA patients.

## Methods

### Study population

This study included 105 patients who were newly diagnosed with RA (these patients without medicaments and with symptom duration of fewer than 12 months) and who were admitted to the Department of Rheumatology and Immunology of the Second Affiliated Hospital of Xi’an Jiaotong University during the period from October 2017 to February 2020. All the patients fulfilled the 2010 American College of Rheumatology criteria for RA^23^. Patients who had other autoimmune diseases, hematologic diseases, malignancies, infections, or had any history of other chronic diseases such as diabetes mellitus, dyslipidemia, thyroid dysfunction, severe liver or kidney impairment as well as those receiving corticosteroids treatment within the last 3 months were excluded. One hundred and two healthy subjects were recruited from the health examination center of our hospital. All the medical examinations of healthy controls were normal. Healthy controls had no current or past history of cardiovascular, metabolic, inflammatory or neoplastic disease. Additionally, healthy controls showed age and gender distributions similar to those of the RA patients in the study. The study was approved by the Research Committee of Human Investigation of Xi'an Jiaotong University Health Science Center and all subjects provided written informed consent. All methods were carried out in accordance with relevant guidelines and regulations.

### Assessment of disease activity

According to the Disease Activity Score in 28 joints based on C-reactive protein (DAS 28-CRP)^24^, RA patients can be described as low (DAS 28-CRP ≤ 2.7), moderate (2.7 < DAS 28-CRP ≤ 4.1) or high (DAS 28-CRP > 4.1) activity, respectively. We define patients with moderate and high disease activity as active RA, whereas the other group of patients with DAS 28-CRP less than or equal to 2.7 were defined as low activity RA.

### Clinical and laboratory parameters

Patients’ characteristics, including age, gender, medical history, symptoms and signs, diagnosis, treatment, laboratory testing results were collected from their electronic medical records. Blood samples were obtained on the day of admission and were sent to our hospital’s clinical laboratory for testing plasma FIB, d-dimer, FDP, and other indices.

### Measurement of plasma FIB, d-dimer and FDP

The soluble plasma FIB is converted into its insoluble polymer fibrin by the enzyme thrombin. The clotting time for diluted plasma is inversely proportional to the FIB concentration of the plasma. By using this principle, Clauss developed a simple procedure for determining FIB based on measuring the clotting time of diluted plasma after the addition of thrombin^25^. The clotting time obtained in this manner is then compared with that of a standardized FIB preparation. Both plasma d-dimer and FDP were detected by immune turbidimetry. Briefly, d-dimer or FDP in the sample reacts with the anti-d-dimer or anti-FDP mouse monoclonal antibody-coated latex, resulting in agglutination and increase in turbidity. Turbidity changes are then measured using a spectrometer to quantitatively measure the d-dimer or FDP concentration in the sample.

### Statistical analysis

The normality of data distribution was examined by the Kolmogorov–Smirnov test. Continuous variables with the normal distribution were presented as mean values ± standard deviation. Non-normally distributed data were presented as median (interquartile range). Categorical variables were described as frequencies or percentages. The differences of continuous variables were analyzed using The Student's t-test or Mann–Whitney U test, while the chi-square test was performed to compare the differences of categorical variables. Receiver operating characteristic (ROC) curves were plotted to distinguish RA patients from healthy individuals or to differentiate active RA from inactive group. The area under the curve (AUC) and 95% confidence interval (CI) were calculated to assess the diagnostic value of each parameter. The optimal cut-off value, sensitivity, specificity, positive predictive value (PPV), negative predictive value (NPV) and accuracy (AC) of the indices were determined. Spearman's correlation analysis was applied to evaluate the association between variables. Binary logistical regression model was used to identify the independent variables associated with RA disease activity (low activity group VS. active group). After univariate regression analysis, variables with *P* < 0.10 were included in the further forward stepwise regression analysis. Additionally, normality of residual error and multicollinearity were analyzed in logistic regression. Statistical significance was considered as a two-tailed *P* value less than 0.05. All statistical analysis was conducted using SPSS software (version 16.0, Chicago, IL, USA).

## Results

### Study population

Clinical and laboratory characteristics of RA patients and healthy controls are shown in Table 1. Comparable results of clinical characteristics between RA patients and Control, and between the two RA subgroups are shown in Table 2. There were no differences in age and gender distribution, body mass index (BMI) and white blood cells (WBC) between the two groups. Besides, red blood cells (RBC) and hemoglobin (Hb) in RA patients were significantly lower than that in the control group (*P* < 0.001). Age, gender, BMI, disease duration, WBC and anti-CCP were similar between active and low activity RA groups. The active RA had higher levels of ESR, CRP and RF compared with those in low activity RA groups (*P* < 0.001 or *P* < 0.05). But active RA presented reduced levels of RBC and Hb relative to low activity RA groups.

**Table 1:** Clinical characteristics of control and RA patients including the two RA subgroups.

	RA (*N* = 105)	Control (*N* = 102)	Low activity RA (*N* = 52)	Active RA (*N* = 53)
Age (years)	55.26 ± 16.27	53.69 ± 14.95	53.28 ± 19.00	56.98 ± 13.64
Gender (F/M)	78/27	76/26	39/13	39/14
BMI (kg/m^2^)	25.2 ± 2.7	25.8 ± 3.2	25.5 ± 3.1	24.7 ± 2.3
CRP (mg/L)	19.48 (7.21–50.62)	1.9 (0.7–2.9)	10.95 (5.87–16.9)	30.30 (10.56–53.90)
ESR (mm/h)	43.0 (17–76)	5.61 (2.93–10.45)	28.0 (15–62)	57.0 (21–83)
RF (IU/mL)	171.56 (25.2–410.00)	5.02 (2.30–13.40)	131.83 (21.20–310.00)	275.29 (35.16–907.0)
Anti-CCP (U/mL)	273.6 (43.8–395.0)	11.1 (6.2–17.5)	251.3 (20.6–375.0)	306.8 (91.7–400.0)
WBC (10^9^/L)	6.65 ± 3.02	6.09 ± 2.23	6.42 ± 2.82	6.88 ± 3.36
RBC (10^12^/L)	4.09 ± 0.64	4.64 ± 0.62	4.24 ± 0.51	3.93 ± 0.46
Hb (g/L)	118.48 ± 20.77	137.20 ± 18.28	123.12 ± 20.38	110.37 ± 17.95
PLT (10^9^/L)	235.12 ± 82.71	202.63 ± 76.27	216.05 ± 84.98	248.87 ± 89.13
FIB (mg/dL)	370.40 ± 126.02	274.73 ± 73.39	315.58 ± 96.44	401.76 ± 122.10
d-dimer (µg/L)	2962.78 ± 1892.94	907.13 ± 545.65	2145.61 ± 975.12	4382.46 ± 1881.72
FDP (mg/L)	19.43 ± 12. 31	2.15 ± 1.03	5.17 ± 3.48	37.13 ± 13.44

Patients in low activity group with a DAS 28-CRP score lower than 2.7; active patients with a DAS 28-CRP score of 2.7 and higher.

*DAS28-CRP* Disease Activity Score in 28 joints based on C-reactive protein, *F* female, *M* male, *BMI* body mass index, *CRP* C-reactive protein, *ESR* erythrocyte sedimentation rate, *RF* rheumatoid factor, *Anti-CCP* anti-cyclic citrullinated peptide, *WBC* white blood cells, *RBC* red blood cells, *Hb* hemoglobin, *PLT* platelet, *FIB* fibrinogen, *FDP* fibrin degradation product.

**Table 2:** Comparison of clinical characteristics between RA patients and Control, and between the two RA subgroups.

	*P*-value (RA vs. control)	*P*-value (low activity RA vs. active RA)
Age (years)	0.117	0.236
Gender (F/M)	0.971	0.868
BMI (kg/m^2^)	0.20	0.45
CRP (mg/L)	< 0.001	< 0.001
ESR (mm/h)	< 0.001	< 0.001
RF (IU/mL)	< 0.001	< 0.05
Anti-CCP (U/mL)	< 0.001	0.373
WBC (10^9^/L)	0.139	0.480
RBC (10^12^/L)	< 0.001	< 0.01
Hb (g/L)	< 0.001	< 0.05
PLT (10^9^/L)	< 0.01	< 0.01
FIB (mg/dL)	< 0.001	< 0.001
d-dimer (µg/L)	< 0.001	< 0.001
FDP (mg/L)	< 0.01	< 0.001

Patients in low activity group with a DAS 28-CRP score lower than 2.7; active patients with a DAS 28-CRP score of 2.7 and higher.

*DAS28-CRP* Disease Activity Score in 28 joints based on C-reactive protein, *F* female, *M* male, *BMI* body mass index, *CRP* C-reactive protein, *ESR* erythrocyte sedimentation rate, *RF* rheumatoid factor, *Anti-CCP* anti-cyclic citrullinated peptide, *WBC* white blood cells, *RBC* red blood cells, *Hb* hemoglobin, *PLT* platelet, *FIB* fibrinogen, *FDP* fibrin degradation product.

### Plasma levels of FIB, d-dimer and FDP

As shown in Tables 1 and 2, plasma levels of FIB, d-dimer and FDP were increased in RA patients compared with healthy controls (*P* < 0.001 or *P* < 0.01). Patients with active RA showed higher levels of plasma fibrin, d-dimer and FDP in comparison to low activity RA (*P* < 0.001).

**Figure 1:**
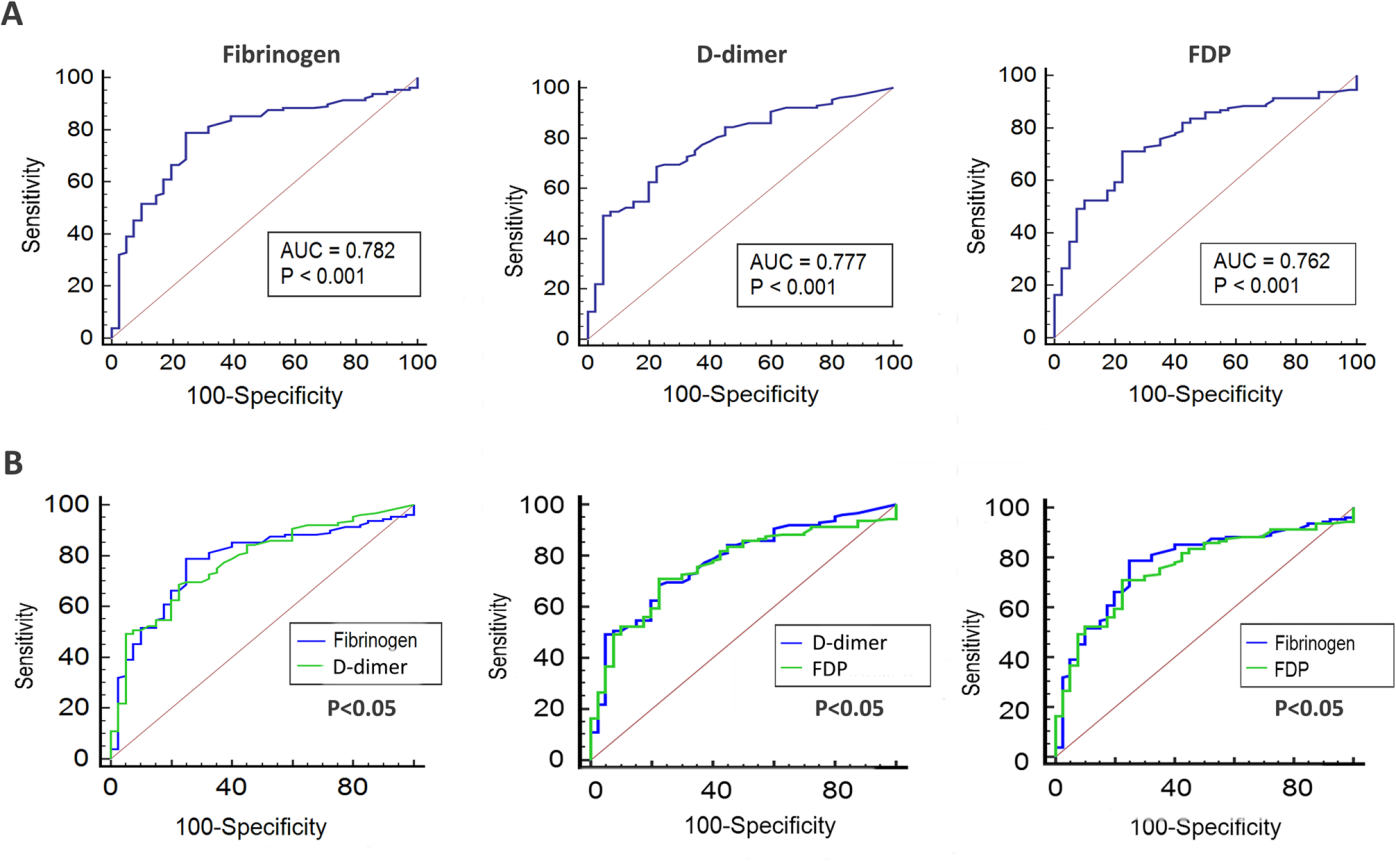
Performance of plasma fibrinogen, d-dimer, and FDP in discriminating RA patients from healthy controls. (**A**) The ROC curves of the three markers in differentiating RA patients from healthy controls. (**B**) Comparison of ROC curves among the three markers. *ROC* receiver operating characteristic curve, *FDP* fibrin degradation product.

In order to evaluate the diagnostic performance of plasma fibrin, d-dimer and FDP, we further applied ROC curve analysis. As shown in Table 3, for RA patients versus healthy controls, the AUC of FIB, d-dimer and FDP was 0.782, 0.777 and 0.762, respectively (all *P* < 0.001). No significant difference in the AUC of three markers for distinguishing between RA patients and the healthy controls (Fig. 1, *P* > 0.05).

**Table 3:** The diagnostic value of FIB, d-dimer and FDP for RA.

Parameters	AUC	95% CI	Optimal cut-off value	Specificity (%)	Sensitivity (%)	PPV (%)	NPV (%)	AC (%)
**RA vs. control**								
FIB (mg/dL)	0.782	0.712–0.842	> 286	75.61	78.91	76.39	78.19	77.26
d-dimer (μg/L)	0.777	0.706–0.837	> 470	77.50	68.75	75.34	77.50	73.13
FDP (mg/L)	0.762	0.691–0.825	> 1.45	77.50	71.09	75.96	72.83	74.30
**Active vs. low activity RA**								
FIB (mg/dL)	0.755	0.666–0.830	> 390	82.35	57.81	66.12	76.61	70.08
d-dimer (μg/L)	0.825	0.743–0.889	> 1230	88.24	65.62	71.96	84.80	76.93
FDP (mg/L)	0.793	0.708–0.863	> 1.81	66.67	81.25	78.05	70.91	73.96
CRP (mg/L)	0.732	0.642–0.810	> 5.41	84.31	67.69	72.29	81.18	76.00
ESR (mm/h)	0.588	0.428–0.736	> 10	86.75	31.25	55.79	70.22	59.00

*AUC* area under curve, *95% CI* 95% confidence interval, *PPV* positive predictive value, *NPV* negative predictive value, *AC* accuracy, *FIB* fibrinogen, *FDP* fibrin degradation product, *CRP* C-reactive protein, *ESR* erythrocyte sedimentation rate.

**Figure 2:**
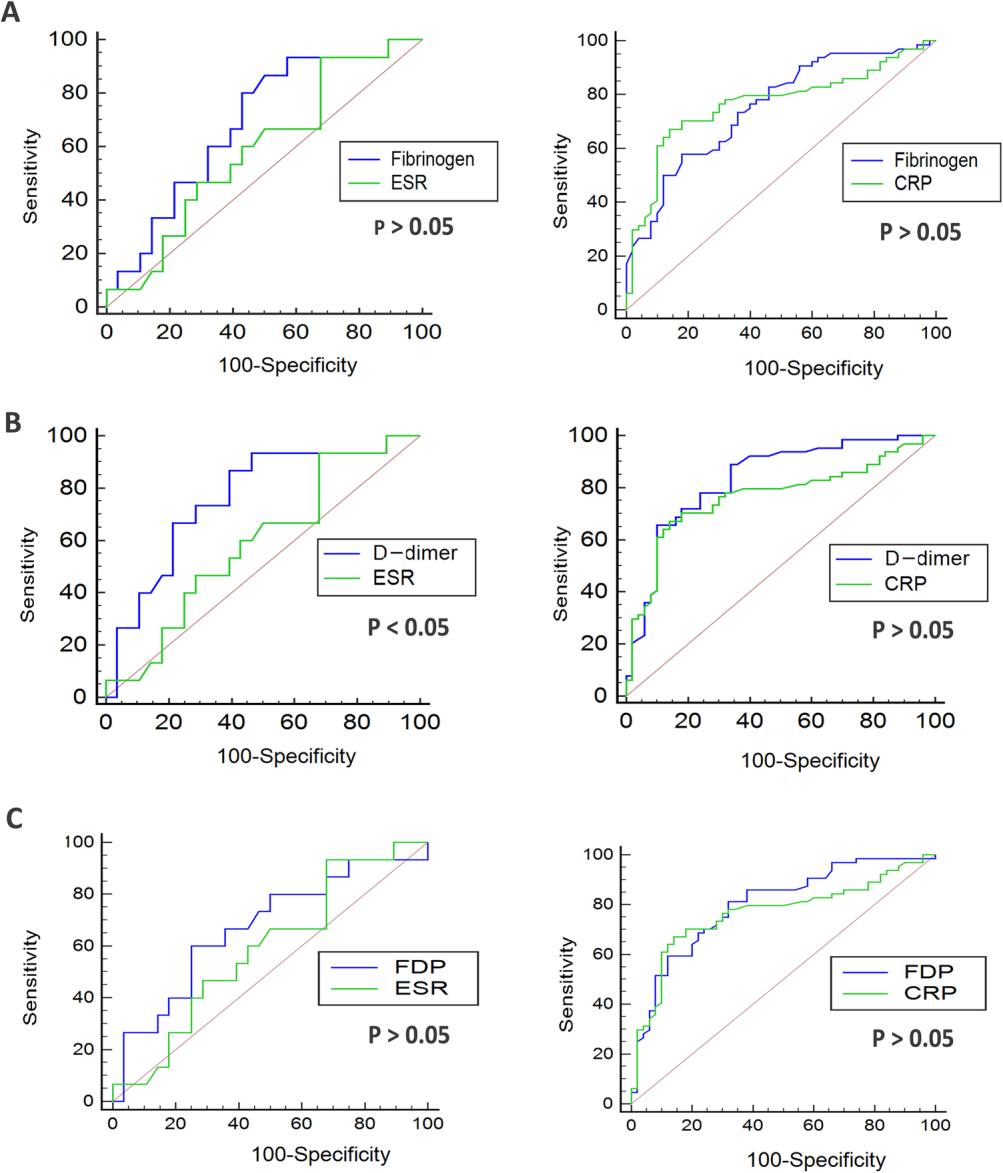
Performance of plasma fibrinogen, d-dimer, and FDP in discriminating active RA from low activity RA. Comparison of ROC curves between fibrinogen (**A**), d-dimer (**B**) or FDP (**C**) and other parameters including ESR and CRP in differentiating active RA from low activity RA. *ROC* receiver operating characteristic curve, *FDP* fibrin degradation product, *ESR* erythrocyte sedimentation rate, *CRP* C-reactive protein.

Table 3 showed the ROC curve analysis for active and low activity RA patients. The AUC of FIB, d-dimer and FDP was 0.755, 0.825 and 0.793, respectively (all *P* < 0.001). The optimal cutoff values of FIB, d-dimer and FDP to differentiate active RA from low activity RA was 390 mg/dL, 1230 μg/L, and 1.81 mg/L, respectively. As shown in Fig. 2, d-dimer had a higher AUC relative to ESR, RF and anti-CCP in distinguishing between active RA and low activity group (*P* < 0.05, *P* < 0.001 and *P* < 0.001, respectively). FDP had a higher AUC relative to RF and anti-CCP while FIB had a higher AUC to anti-CCP in distinguishing between active RA and low activity group (*P* < 0.01, *P* < 0.001 or *P* < 0.05).

### Correlation of plasma coagulation-related markers with disease activity

To further evaluate the relationship between plasma coagulation-related markers and disease activity, we conducted a correlation analysis between plasma levels of these markers and disease activity index DAS-28 CRP, swollen joint count (SJC), tender joint count (TJC), and laboratory parameters including CRP and ESR.

As shown in Table 4, FIB was positively associated with DAS-28 CRP (r = 0.422, *P* < 0.001), CRP (r = 0.557, *P* < 0.001), ESR (r = 0.502, *P* < 0.001), SJC (r = 0.528, *P* < 0.001) and TJC (r = 0.513, *P* < 0.001). d-dimer was positively correlated with DAS-28 CRP (r = 0.490, *P* < 0.001), CRP (r = 0.647, *P* < 0.001), ESR (r = 0.619, *P* < 0.001), SJC (r = 0.635, *P* < 0.001) and TJC (r = 0.629, *P* < 0.001). FDP was positively related with DAS-28 CRP (r = 0.491, *P* < 0.001), CPR (r = 0.611, *P* < 0.001), ESR (r = 0.568, *P* < 0.001), SJC (r = 0.617, *P* < 0.001) and TJC (r = 0.532, *P* < 0.001). Notably, the correlation between d-dimer and disease activity parameters such as CRP and ESR was the strongest.

**Table 4:** Correlation of FIB, d-dimer and FDP with disease activity in RA patients.

Parameters	FIB (mg/dL)		d-dimer (μg/L)		FDP (mg/L)	
	r	*P*-value	r	*P*-value	r	*P*-value
CRP (mg/L)	0.557	< 0.001	0.647	< 0.001	0.611	< 0.001
ESR (mm/h)	0.502	0.001	0.619	< 0.001	0.568	< 0.001
DAS28-CRP	0.422	< 0.001	0.490	< 0.001	0.491	< 0.001
SJC	0.528	< 0.001	0.635	< 0.001	0.617	< 0.001
TJC	0.513	< 0.001	0.629	< 0.001	0.532	< 0.001

*CRP* C-reactive protein, *ESR* erythrocyte sedimentation rate, *DAS28-CRP* Disease Activity Score in 28 joints based on C-reactive protein, *FIB* fibrinogen, *FDP* fibrin degradation product, *SJC* swollen joint count, *TJC* tender joint count.

### Binary logistic regression analysis of factors independently associated with disease activity in RA patients

In addition, we performed logistic regression analysis to identify the independent variables associated with RA disease activity. After univariate regression analysis, d-dimer and FDP were used for further multivariate regression analysis (*P* < 0.10). Results from forward stepwise regression analysis showed that only d-dimer is an independent variable associated with RA disease activity (OR 2.862, 95% CI (1.851–5.426), *P* < 0.001) (Table 5).

**Table 5:** Logistic regression analysis on the disease activity in RA patients.

Predictors	Univariate regression analyses			Multivariate regression analyses		
	β	OR (95% CI)	*P*-value	β	OR (95% CI)	*P*-value
Gender	0.282	1.326 (0.109–16.133)	0.825			
Age	0.414	0.661 (0.251–1.740)	0.602			
Duration of RA	0.600	1.823 (0.243–13.673)	0.559			
ESR	1.170	3.158 (1.018–8.923)	0.135			
CRP	1.583	4.871 (1.306–11.463)	0.027	0.417	1.518 (0.938–2.457)	0.089
RF	0.367	1.443 (0.438–4.756)	0.547			
Anti-CCP	0.410	1.043 (0.474–2.293)	0.231			
FIB	0.969	1.580 (0.785–5.462)	0.282			
d-dimer	2.931	8.752 (2.198–33.207)	0.012	1.052	2.862 (1.851–5.426)	0.001
FDP	1.268	0.114 (0.015–1.112)	0.093	0.152	0.859 (0.351–2.109)	0.740

*OR* odds ratio, *95% CI* 95% confidence interval, *ESR* erythrocyte sedimentation rate, *CRP* C-reactive protein, *RF* rheumatoid factor, *Anti-CCP* anti-cyclic citrullinated peptide, *FIB* fibrinogen, *FDP* fibrin degradation product.

## Discussion

This study showed that RA patients had higher levels of FIB, d-dimer and FDP than healthy individuals. FIB, d-dimer and FDP were significantly elevated in patients with active RA than those in low activity group. Additionally, plasma levels of these coagulation markers were positively related with parameters reflecting RA disease activity such as DAS-28 CRP, SJC, TJC, CRP and ESR. These plasma coagulation markers contribute to distinguish between active and low activity RA. Logistic regression analyses illustrated that high d-dimer could be associated with the increased risk of disease activity in RA patients.

FIB is an important glycoprotein present in human blood plasma and it is involved in many physiological processes such as wound healing, tissue regeneration and regulation of inflammatory responses^26^. Moreover, FIB has been shown as an important determinant of inflammatory arthritis through its effects on proinflammatory pathways such as NF-κB signaling^27^. It has been demonstrated that the multiple regions of the FIB molecule could bind to CD11b/CD18 integrin receptor expressed on cells associated with the innate immune system such as circulating monocytes, tissue-specific macrophages, and so on^28,29^. Emerging evidence from in vitro studies and animal models implicated FIB in the pathogenesis of RA. For example, an in vitro study showed that treatment of synovial fibroblasts with FIB was followed by IL-8 secretion and upregulation of ICAM-1 expression, resulting in increased adhesiveness of lymphocytes^27^. Another study using collagen-induced arthritis model revealed that mice lacking FIB or missing only CD11b/CD18 integrin receptor-binding domain had fewer arthritic joints compared with the control mice^30^. A recent study showed that circulating levels of FIB are elevated in RA and correlated with markers of inflammation^31^. In this study, the finding of a significant association between plasma levels of FIB and RA disease activity may be a consequence of increased synovialis and articular cartilage injury accompanying with increased FIB concentration in RA patients.

FDP and d-dimer are derived from the fibrin clot breakdown by plasmin. As traditional markers of fibrinolysis, FDP and d-dimer have been analyzed for monitoring venous thromboembolism and preoperative coagulation function. Recently there are several studies reporting that FDP and d-dimer levels were elevated in the setting of systemic inflammation and infection^26,32,33^. A previous study reported that the fluctuation of serum d-dimer is more rapid than serum CRP and ESR in patients with periprosthetic joint infection and the detection of serum d-dimer might be applied effectively in the early diagnosis of postoperative infection^34^. Ma et al*.* demonstrated that plasma levels of d-dimer were associated with disease activity of ANCA-associated vasculitis^35^. A prospective study performed on patients with chronic spontaneous urticaria has revealed that patients with autoimmune status had higher levels of plasma d-dimer than those without autoimmunity^36^. Weinberg et al*.* reported that there were higher levels of d-dimer in the synovium of RA patients than that in patients with osteoarthritis and traumatic joint injury^37^. Although previous studies have shown that the d-dimer level was significantly higher in RA patients than that in controls, little is known about the relationship between plasma levels of FDP and d-dimer and the disease activity in RA patients. A previous study indicated that d-dimer could more accurately reflect the disease activity and prognosis as compared to clinical features in patients with systemic juvenile idiopathic arthritis^38^. Consistent with this study, we found that plasma levels of FDP and d-dimer were significantly related to the severity of RA patients. Moreover, in our study, high level of plasma d-dimer was identified to be as an independent variables associated with RA disease activity by the regression analysis. Notably, both d-dimer and soluble CD40 ligand (sCD40L) are soluble platelet activation markers. Recent study revealed that sCD40L was increased in RA patients and its level was associated with the disease activity DAS28^39^. CD40/CD40L costimulatory pathway contributes to the overactivation and proliferation of autoreactive CD4^+^ T cells, which maintains autoantibody production and inflammation in RA^39^. However, there was no study reporting the association between d-dimer and activation of CD4^+^ T cells in RA. In our further study, we will evaluate whether plasma levels of d-dimer is correlated with the number and activation of CD4^+^ T cells in RA patients. Given that d-dimer is regarded as a marker of the platelet activation, then we also expected to clarify whether d-dimer and platelets were implicated in the pathogenesis of RA in our further study. This will help us make a better understanding of the pathogenic mechanisms of RA.

This study has some limitations. Firstly, this study was a single center study and has the relatively low number of patients. Secondly, this study was a retrospective study, which could not be used for explicating the causality between coagulation related markers and the disease activity of RA patients. The prospective studies of large-scale and multi-center are required to confirm our findings and to clarify the mechanism underlying the relationship between these markers and RA. In summary, this study systematically investigated the role of FIB, FDP and d-dimer as biomarkers in determining the disease activity of RA. We found that plasma FIB, d-dimer, and FDP were increased in RA patients and positively correlated with RA disease activity. d-dimer was revealed as an independent variable associated with the disease activity in RA patients. This study indicated that d-dimer may be an effective biomarker for indicting RA disease activity.

## Abbreviations

RA: Rheumatoid arthritis
BMI: Body mass index
DAS28-CRP: Disease Activity Score in 28 joints based on C-reactive protein
CRP: C-reactive protein
ESR: Erythrocyte sedimentation rate
RF: Rheumatoid factor
Anti-CCP: Anti-cyclic citrullinated peptide
WBC: White blood cells
RBC: Red blood cells
Hb: Hemoglobin
PLT: Platelet
FIB: Fibrinogen
FDP: Fibrin degradation product
ROC: Receiver operating characteristic
AUC: Area under curve
95% CI: 95% Confidence interval
PPV: Positive predictive value
NPV: Negative predictive value
AC: Accuracy
